# Chromosomal organization of four classes of repetitive DNA sequences in killifish *Orestias ascotanensis* Parenti, 1984 (Cyprinodontiformes, Cyprinodontidae)

**DOI:** 10.3897/CompCytogen.v11i3.11729

**Published:** 2017-07-25

**Authors:** Cristian Araya-Jaime, Natalia Lam, Irma Vila Pinto, Marco A. Méndez, Patricia Iturra

**Affiliations:** 1 Facultad de Medicina, Universidad de Chile, ICBM, Programa de Genética Humana, Casilla 70061, Santiago, Chile; 2 Departamento de Producción Animal, Facultad de Ciencias Agronómicas, Universidad de Chile. Santa Rosa 11315, La Pintana, Santiago, Chile; 3 Departamento de Ciencias Ecológicas, Facultad de Ciencias, Universidad de Chile, Las Palmeras 3425, Casilla 653, CP 780-0024, Santiago, Chile

**Keywords:** *Orestias*, molecular cytogenetics, multigene families

## Abstract

*Orestias* Valenciennes, 1839 is a genus of freshwater fish endemic to the South American Altiplano. Cytogenetic studies of these species have focused on conventional karyotyping. The aim of this study was to use classical and molecular cytogenetic methods to identify the constitutive heterochromatin distribution and chromosome organization of four classes of repetitive DNA sequences (histone H3 DNA, U2 snRNA, 18S rDNA and 5S rDNA) in the chromosomes of *O. ascotanensis* Parenti, 1984, an endemic species restricted to the Salar de Ascotán in the Chilean Altiplano. All individuals analyzed had a diploid number of 48 chromosomes. C-banding identified constitutive heterochromatin mainly in the pericentromeric region of most chromosomes, especially a GC-rich heterochromatic block of the short arm of pair 3. FISH assay with an 18S probe confirmed the location of the NOR in pair 3 and revealed that the minor rDNA cluster occurs interstitially on the long arm of pair 2. Dual FISH identified a single block of U2 snDNA sequences in the pericentromeric regions of a subtelocentric chromosome pair, while histone H3 sites were observed as small signals scattered in throughout the all chromosomes. This work represents the first effort to document the physical organization of the repetitive fraction of the *Orestias* genome. These data will improve our understanding of the chromosomal evolution of a genus facing serious conservation problems.

## Introduction

Cytogenetic analysis is a useful tool for describing evolutionary patterns and the histories of closely-related species or species complexes. *Orestias* Valenciennes, 1839 is a genus of freshwater fish endemic to the South American Altiplano. The genus includes 45 species, grouped into four complexes: *O. cuvieri*, *O. mulleri*, *O. gilsoni* and *O. agassii* (Costa 1999, Parenti 1984). Conventional karyotyping studies involving the seven species of *O. agassii* complex found in the Chilean Altiplano (17°and 22°S) have revealed variations in the chromosome number (2n=48-55) and the presence/absence of microchromosomes, suggesting that Robertsonian rearrangements may play a role in the karyotypic evolution of these species (Arratia 1982, Vila et al. 2007, 2010, 2011, Habit et al. 2006, Villwock and Sienknecht 1996).

The most commonly-used approaches for comparative cytogenetic analysis of fish include characterizing the distribution and composition of constitutive heterochromatin and fluorescence *in situ* hybridization (FISH) mapping of molecular landmarks such as 18S and 5S ribosomal DNA. New markers of repeated elements such as histone H1, H3 and H4 genes and the U2 snRNA gene have recently been incorporated into these studies (Hashimoto et al. 2011, Utsunomia et al. 2014a, Silva et al. 2015, Utsunomia et al. 2016). The repetitive nature of these sequences makes them useful markers for chromosomal mapping as they provide insight into the structure and organization of the genome and facilitate detection of karyotype rearrangements (Kavalco et al. 2013). However, studies involving chromosomal mapping of repetitive sequences in fish are scarce and typically focus exclusively on the location of ribosomal DNA sites. Studies involving physical mapping of histone genes and mobile elements are also limited, and data is available for only a few species (Pendas et al. 1994, Hashimoto et al. 2011, Ferreira et al. 2011).

*Orestias ascotanensis* Parenti, 1984 is an endemic species restricted to the small isolated freshwater springs of the Salar de Ascotán. This fish is on the Chilean Endangered Species List (MINSEGPRES, 2008). Major threats to conservation of this species include global climate change and intense regional mining activity. Both situations contribute to a gradual lowering of the water level in the springs, potentially making the salinity of the water incompatible with life for these populations (Vila et al. 2007, Morales et al. 2011). The *O. ascotanensis* karyotype consists of 48 chromosomes, which is the most common diploid number among species in the order Cyprinodontiformes. The chromosomal formula is (2M + 4SM + 4 ST + 38T) (Vila et al. 2010).

The aim of this study was to identify for the first time the constitutive heterochromatin distribution and chromosome organization of four classes of repetitive DNAs (histone H3 DNA, U2 snRNA and 18S and 5S rDNA) in the chromosomes of *O. ascotanensis*. This data will shed light on the physical organization of the repetitive fraction of the genome of *O. ascotanensis*, a species endemic to the Chilean Altiplano that is facing serious conservation problems. In addition, application of these cytogenetic tools will allow for comparisons among *Orestias* species, facilitating the identification of genomic modifications underlying the chromosomal variations observed in these species.

## Materials and methods

### Sampling and mitotic chromosome isolation

Eight *O. ascotanensis* individuals, 3 male and 5 female, were obtained from Salar de Ascotán (21°31'S 68°15'W), Region de Antofagasta, Chile, under Scientific Collection Permit Number 1103 issued by SERNAPESCA. The fish were transported to the laboratory and maintained alive in aquaria until processing. Mitotic chromosomes were obtained from kidney cell suspensions according to a modified version of the protocol established by Foresti et al. (1993). Approximately 20 metaphase spreads from different individuals were analyzed to confirm the diploid number and karyotype structure of *O. ascotanensis*. The chromosomes were measured and classified as metacentric (m), submetacentric (sm), subtelocentric (st) or telocentric (t) (Levan et al. 1964), and the karyotype was arranged according to Vila et al. (2010). The images were captured with a digital camera (Nikon D60) attached to an epifluorescence photomicroscope (Nikon Optiphot). Karyotype mounting and image brightness and contrast adjustments were performed in Adobe Photoshop CS6.

### Chromosome banding: C- banding and *CMA*_3_

The constitutive heterochromatin (HC) distribution pattern was visualized according to a modified version of the protocol established by Sumner (1972); briefly, chromosomes were subjected to hydrolysis with HCL 0.2 N for 45 min at room temperature, denatured with 5% barium hydroxide at 60°C for 8 min and incubated in saline buffer 2× SSC, and stained with propidium iodide (50 ug/mL) (Lui et al. 2009). Chromomycin A_3_ staining was then performed using the method described by Sola et al. (1992). Metaphase plates were observed using a Nikon (Optiphot) microscope with the appropriate filter.

### Repetitive sequence probes and FISH experiments

U2 snRNA, 5S rDNA, 18S rDNA and histone H3 DNA probes were obtained from the genomic DNA of *O. ascotanensis*. DNA was collected from a piece of fin tissue with the Wizard Genomic DNA Purification Kit (Promega) according to manufacturer instructions, using previously-described primers (Table 1). The U2 snRNA and 5S rDNA probes were labeled by PCR with biotin-16-dUTP, and the 18S rDNA and histone H3 DNA probes were labeled by PCR with digoxigenin-11-dUTP. FISH was performed under high-stringency conditions using the method described by Pinkel et al. (1986). Slides were incubated with RNAse (50μg/ml) for 1 h at 37°C. Next, the chromosomal DNA was denatured in 70% formamide/2× SSC for 5 min at 70°C, and the slides were taken through an ice-cold ethanol series (70°-80°-100°). For each slide, 30μl of hybridization solution containing 200 ng of each labeled probe, 50% formamide, 2× SSC and 10% dextran sulfate was denatured for 10 min at 95°C, dropped onto the slides and hybridized overnight at 37°C in a 2× SSC moist chamber. After hybridization, slides were washed in 0.2× SSC/15% formamide for 20 min at 42°C, followed by a second wash in 0.1× SSC for 15 min at 60°C and a final wash at room temperature in 4× SSC/0.5% Tween for 10 min. Probe detection was carried out with Avidin-FITC (Sigma) or anti-digoxigenin-rhodamine (Roche). Chromosomes were counterstained with DAPI (4’,6-diamidino-2-phenylindole, Vector Laboratories).

**Table 1.** Primers used to PCR amplification for gene fragments 5S rDNA, 18S rDNA, U2 snRNA and Histone H3.

Gene	Primers sequences	References
5S rDNA	5SA 5’-TCAACCAACCACAAAGACATTGGCAC-3’	Pendás et al. 1994
	5SB 5’-TAGACTTCTGGGTGGCCAAAGGAATCA-3’	
18S rDNA	18SF 5’-GTAGTCATATGCTTGTCTC-3’	White et al. 1990
	18SR 5’-TCCGCAGGTTCACCTACGGA-3’	
U2snRNA	U2 F 5’-ATCGCTTCTCGGCCTTATG-3’	Bueno et al. 2013
	U2 R 5’-TCCCGGCGGTACTGCAATA-3’	
Histone H3	H3F 5’- ATATCCTTRGGCATRATRGTGAC-3’	Colgan et al. 1998
	H3R 5’- ATGGCTCGTACCAAGCAGACVGC-3’	

## Results

**Figure 1.**
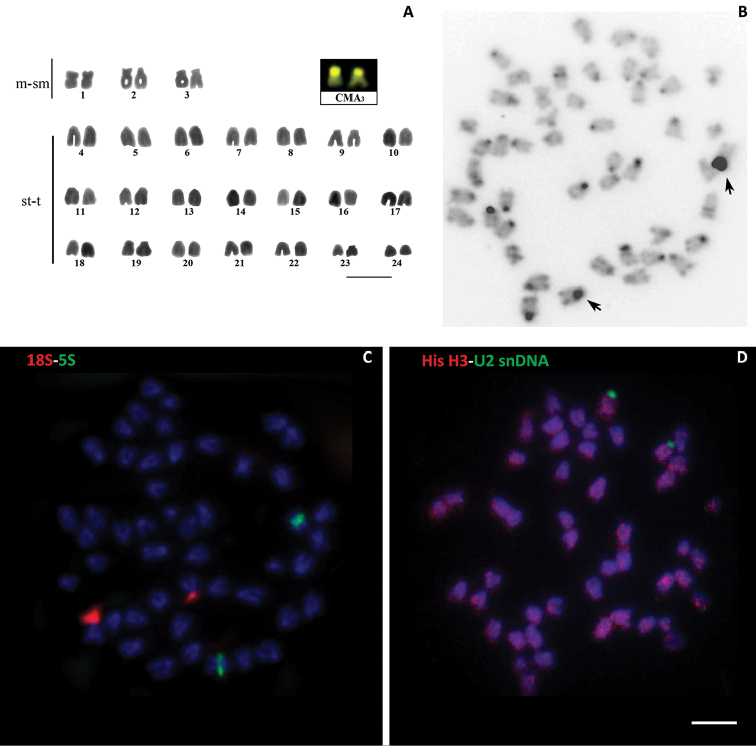
Karyotype of *O. ascotanensis* (female), 2n=48. A Giemsa-stained karyotype and CMA_3_ positive bands (box) B C-banded somatic metaphase C metaphases counterstained with DAPI after FISH treatment using 5S and 18S rDNA probes D metaphases counterstained with U2 snDNA/histone H3 DNA probes. The arrows show a block of HC on the short arm of pair 3. Bar = 10µm.

All *O. ascotanensis* individuals analyzed had a diploid number of 48 chromosomes, consistent with the chromosome formula defined by Vila et al. (2010) (Fig. 1A). No morphologically differentiated sex chromosomes were found when metaphase plates from males and females were compared. C-banding revealed that the constitutive heterochromatin was mainly distributed in the pericentromeric regions of most chromosomes (Fig. 1B). Submetacentric pair 3 was noteworthy due to the presence of conspicuous HC blocks extending along the entire short arm. An interstitial C-band was present on the long arm of chromosome pair 2, proximal to the centromeric region. Additionally, in all observed metaphases, CMA_3_-banding revealed that the short arm of pair 3 was strongly stained, reflecting a greater abundance of GC bases in this heterochromatic region (see box, Fig. 1A).

Dual FISH detected 18S and 5S rDNA probes on different chromosome pairs (Fig. 1C). The major rDNA cluster (18S) was located on the short arm of pair 3, with a size polymorphism between the bearing arms of these sequences. 5S rDNA sequences were detected in the region proximal to the centromere of the long arm of pair 2, coincident with the HC band described above. Dual FISH (Fig. 1D) identified a single block of U2 snDNA sequences in the pericentromeric region of a subtelocentric chromosome pair, while histone H3 sites were detected as scattered signals throughout the *O. ascotanensis* chromosomes.

## Discussion

Previous cytogenetic studies involving the seven species of *O. agassii* complex of the Chilean Altiplano were limited to characterizing the chromosome number and morphology of the species. The diploid number has been reported to vary between 48 and 55 chromosomes and the fundamental number of chromosome arms (FN) between 54 and 56 (Arratia 1982, Vila et al. 2010, Vila et al. 2011)

Characterization of the repetitive fraction of the genome is a useful tool for identifying recent genomic changes during the evolutionary process as well as possible hotspots associated with chromosomal rearrangements (Valente et al. 2011, Ozouf-Costaz et al. 2004, Yano et al. 2014). The organization of the repetitive fraction of the genome in Cyprinodontiformes fish has remained relatively unexplored, with prior studies focusing primarily on the distribution and composition of constitutive heterochromatin and physical mapping of 18S rDNA genes. Noteworthy studies include reports on: *Fundulus* (Lacépède, 1803) (Kornfield 1981); *Austrolebias* Costa, 1998 (García et al. 1993, 1995, 2001, 2014, 2015); *Aphanius* Nardo, 1827 (Vitturi et al. 2005, Gaffaroglu et al. 2014) and *Hypsolebias* Costa, 2006 (Do Nascimento et al. 2014), with constitutive heterochromatin found to be distributed mainly in centromeric, telomeric and interstitial regions. In addition, in some species of *Chromaphyosemion* Myers, 1924, conspicuous blocks of HC have been identified in the short arm of bi-armed chromosomes (Völker et al. 2005, Völker et al. 2006, Volker et al. 2007, Völker et al. 2008).

In *O. ascotanensis*, the C-band regions were found mainly in the pericentromeric regions, unlike other Cyprinodontiformes that have been studied. CMA_3_ also revealed that the conspicuous blocks of HC found in the short arm of pair 3 have a higher proportion of GC bases than previously-analyzed fish. Moreover, the presence of 18S rDNA sequences in this chromosome arm defines this pair as the carrier of the NOR. An association between 18S and 28S rDNA sequences and heterochromatin has been found in other fish, such as salmonids (Fujiwara et al. 1998, Pendas et al. 1994), species of the genera *Epinephelus* Bloch, 1793 (Sola et al. 2000), *Imparfinis* Eigenmann & Norris, 1900 and *Pimelodella* Eigenmann & Eigenmann, 1888 (Gouveia et al. 2013) and sturgeon species (Fontana et al. 2003), suggesting that the repeated HC sequences play an important role and exercise diverse functions in the eukaryotic genome (Grewal and Jia 2007). It has even been postulated that heterochromatin is involved in maintaining the structure of the nucleolus and the integrity of ribosomal DNA repeats (McStay and Grummt 2008).

The single 18S rDNA sequence-bearing chromosome pair in *O. ascotanensis* (Fig. 1C) is a feature observed in most teleosts (Pisano and Ghigliotti 2009, Gornung 2013). However, varied numbers of chromosomes carrying the major ribosomal DNA cluster have been reported in Cyprinodontiformes, with findings ranging from one to seven pairs of chromosomes (Völker et al. 2005, Völker et al. 2006, Volker et al. 2007, Völker et al. 2008). Data on the chromosomal location of the minor ribosomal sites are almost non-existent for Cyprinodontiformes. In *O. ascotanesis*, pair 2 is the 5S-bearing pair, with submetacentric morphology (Fig. 1C). The hybridization signal was detected on the long arm, proximal to the centromere region, associated with the interstitial heterochromatic band of this pair. 5S and 18S rDNA are typically localized on different chromosomes in vertebrates, including teleosts (Scacchetti et al. 2015, Sánchez-Romero et al. 2015). However, in the Cyprinodontiform *Lebias fasciata* (Valenciennes, 1821), FISH mapping has shown that the 28S and 5S ribosomal DNA probes co-localize on a pair of telocentric chromosomes, conserving the 5S locus on the medial position of the chromosome (Tigano et al. 2004). In general, these sequences vary among teleosts in relation to the chromosomal distribution due to their association with transposable elements, typically within the internal spacer regions (Martins and Galetti 2001, Cabral-de-Mello et al. 2011, Scacchetti et al. 2012, Sene et al. 2015).

Data on the physical location of U2 snRNA sites in fish is also scarce. Two general configurations are recognized: (I) clustered on a single pair of chromosomes, as in the present case and (II) scattered throughout the genome (Ubeda-Manzanaro et al. 2010, Utsunomia et al. 2014, Scacchetti et al. 2015, Silva et al. 2015). According to Medrano et al. (1988), teleosts show low levels of genomic compartmentalization, suggesting that the configuration observed for the U2 snRNA, 5S rDNA and 18S rDNA in *O. ascotanensis* represents a relatively simple genomic organization.

The finding of scattered histone H3 sites distributed throughout the *O. ascotanensis* chromosomes diverges strongly from data reported for other fish, such as Characiformes (Hashimoto et al. 2011, Pansonato-Alves et al. 2013a, Silva et al. 2015), Siluriformes (Hashimoto et al. 2013, Pansonato-Alves et al. 2013b) and Perciformes (Lima-Filho et al. 2012), which generally have large blocks of these sequences in specific chromosome pairs. The histone H3 DNA site distribution found in *O. ascotanensis* chromosomes is similar to the organization described for *Synbranchus marmoratus* Bloch, 1795, suggesting that H3 sequences may be organized in small, abundant copies throughout the genome, as has been proposed by Utsunomia et al. (2014b). Further studies are necessary to confirm that this scattered distribution of H3 DNA is conserved among *Orestias* species.

To understand the relationship of these repeated genomic elements to the chromosomal evolution of these fish and to historical changes in the fishes’ environment, further studies are needed to physically map the repetitive DNA in other *Orestias* representatives. These findings would enhance our understanding of native wildlife species facing serious conservation problems.
